# Neuromodulation–inspired gated associative memory networks: extended memory retrieval and emergent multistability

**Published:** 2025-12-15

**Authors:** Daiki Goto, Hector Manuel Lopez Rios, Monika Scholz, Suriyanarayanan Vaikuntanathan

**Affiliations:** 1 Department of Physics, The University of Chicago, Chicago, Illinois 60637, USA; 2 The James Franck Institute, The University of Chicago, Chicago, Illinois 60637, USA^§^; 3 Department of Chemistry, The University of Chicago, Chicago, Illinois 60637, USA; 4 Max Planck Institute for Neurobiology of Behavior—caesar, Bonn 53175, Germany

## Abstract

Classical autoassociative memory models have been central to understanding emergent computations in recurrent neural circuits across diverse biological contexts. However, they typically neglect neuromodulatory agents that are known to strongly shape memory capacity and stability. Here we introduce a minimal, biophysically motivated associative memory network where neuropeptide-like signals are modeled by a self-adaptive, activity-dependent gating mechanism. Using many-body simulations and dynamical mean-field theory, we show that such gating fundamentally reorganizes the attractor structure: the network bypasses the classical spin-glass transition, maintaining robust, high-overlap retrieval far beyond the standard critical capacity, without shrinking basins of attraction. Mechanistically, the gate stabilizes transient “ghost” remnants of stored patterns even far above the Hopfield limit, converting them into multistable attractors. These results demonstrate that neuromodulation-like gating alone can dramatically enhance associative memory capacity, eliminate the sharp Hopfield-style catastrophic breakdown, and reshape the memory landscape, providing a simple, general route to richer memory dynamics and computational capabilities in neuromodulated circuits and neuromorphic architectures.

## I. INTRODUCTION

Recent large-scale measurements of neuronal activity show remarkable collective population dynamics with large populations of neurons cycling repeatedly through brain states [1]. These dynamics reflect internal states of the brain, but can also map to behavioral states [2–5]. How the architecture of the brain sets up such widely coordinated patterns is an area of active research. Specifically, theoretical models with simple connectivity rules have shown the emergence of such patterns. For example, classical autoassociative memory models—exemplified by the Amari–Hopfield recurrent neural networks (RNNs) [6–8]—embed a finite set of patterns directly in the interaction matrix so that the dynamics relax, given partial cues, to fixed-point attractors representing those patterns.

Although simple, these classical autoassociative models have been used to model aspects of neuronal dynamics in living neuronal systems such as the hippocampus [9, 10] and cortex [11]. These models incorporate the pairwise coupling between neurons that mimic biological synapses, but do not incorporate potentially important extrasynaptic signaling contributions. Indeed, extrasynaptic activity due to neuromodulators (biogenic amines or neuropeptides) is ubiquitous and can influence the activity of large neuron populations, often via multiple parallel biochemical pathways [12, 13]. For example, a recently completed identification of the neuropeptidergic connectome of the nematode *Caenorhabditis elegans* [14–16], revealed that the “hard-wired” synaptic network is overlaid by a “wireless” neuropeptidergic network. Beyond nematodes, these signaling modes are increasingly appreciated across other species, including in mice [17] and humans [18]. Broad perturbations of neuromodulators affect behavioral state and state transitions [19–22], as well as learning and memory [23]. In particular, neuropeptide release is typically activity-dependent, and once secreted these molecules act on any nearby neurons expressing relevant receptors. These modulators can alter neuronal dynamics in two distinct ways, either by directly affecting synaptic strength or by altering neuronal properties such as input-output relationships [12]. While synaptic modulation has been widely studied both computationally and experimentally, the effect of neuromodulators on neuronal conductivity has not been assessed. Specifically, how and if neuromodulators affect the capacity of the nervous system for memory retrieval and computability is unknown.

Here, we attempt to address this question by considering a minimal, yet biologically grounded model of neuromodulation. Based on experimental evidence that neuromodulation often acts multiplicatively, effectively gating the neuronal inputs or regulating the integration timescales of individual neurons [19, 24, 25], we consider a minimal model system that operates as a coupled two-layer network: a primary layer of neuronal activity and an auxiliary layer of neuromodulators that multiplicatively regulate the primary dynamics. This concept of multiplicative gating is also canonical in machine learning RNNs. Architectures such as Long Short-Term Memory (LSTM) [26, 27] and Gated Recurrent Unit (GRU) [28, 29] utilize gating specifically to control information flow and emergent timescales. While it has been shown that random gated networks robustly self-organize into a marginally stable state, even in parameter regimes that would otherwise be chaotic [30, 31], the implications of this self-adaptive gating for *associative memory* remain largely unexplored. Using a combination of direct many-body simulations and Dynamical Mean-Field Theory (DMFT), we show that gating fundamentally alters the memory retrieval dynamics. Our main finding is that the gated network bypasses the standard sharp transition to the spin-glass phase, maintaining high-overlap retrieval well beyond the classical critical capacity $\alpha_{\text{c}}$. We identify the microscopic mechanism: adaptive gating slows down the escape from the “ghosts” of stored patterns, eventually stabilizing them into true fixed points. Other instances of autoassociative models with higher-order interactions, known as dense associative memories, have also shown increased storage capacities, algebraically [32–37] or even exponentially large [38–40], but at the expense of narrowed empirical basin sizes [40–42]. Crucially, gating-induced capacity enhancement does not shrink the empirical basins of attraction and allows for a broader range of cues for retrieval. Finally, we reveal how gating can lead to a novel memory retrieval phase of continuous multistability, where the system supports a continuous manifold of stable fixed-point attractors.

The remainder of the paper is organized as follows: In Sec. II, we define the self-adaptively gated associative memory network and discuss its biological and computational motivations, followed by a brief review of the baseline properties of the standard ungated network. In Sec. III, we present the main phenomenological results from direct numerical simulations, including the extended memory retrieval phase. In Sec. IV, we develop the DMFT formalism for the gated network to establish that the findings from finite-size simulations persist in the thermodynamic limit, and we analyze the resulting long-time behavior to characterize steady-state overlaps. In Sec. V, we then visualize and characterize the geometry of the emergent continuous multistable attractors in a simplified system storing only two patterns. Finally, in Sec. VI, we summarize our findings and discuss their implications for both biological systems and neuromorphic computing.

## II. MODEL AND BACKGROUND

### A. Self-adaptively gated associative memory network

**FIG. 1.**
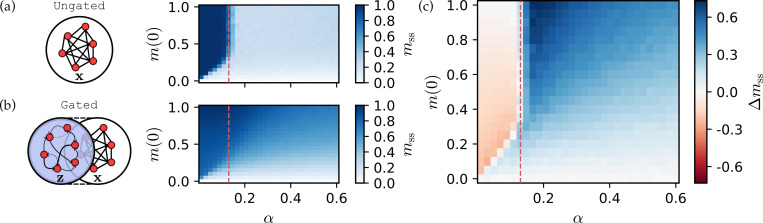
Gating enables memory retrieval beyond the traditional capacity threshold without narrowing empirical attractor basins. (a) Ungated benchmark. (Left) Schematic of the standard Amari–Hopfield network (γ=0). (Right) The steady-state overlap $m_{\text{ss}}$ plotted as a function of memory load α and initial overlap m(0). Color intensity represents the retrieval quality, ranging from failure (white) to successfulæ retrieval (darkblue). The vertical dashed line marks the standard critical capacity $\alpha_{\text{c}}≃0.13$ of the ungated network. Notably, catastrophic blackout (the spin-glass transition) is observed for $\alpha >\alpha_{\text{c}}$. (b) Self-adaptively gated network. (Left) Schematic of the architecture, where an auxiliary neuromodulatory layer z gates the recurrent neural layer x. (Right) Corresponding phase diagram for the same range of parameters in the binary gating limit (γ→∞). In contrast to (a), catastrophic blackout is *not* observed. (c) Performance gain. The difference in steady-state overlaps, $\text{Δ}m_{\text{ss}}=m_{\text{ss}}^{\gamma \to \infty }-m_{\text{ss}}^{\gamma =0}$, highlighting the region where gating recovers memories that fail in the ungated network. This demonstrates that the capacity gain is achieved *without* degrading the size of the attractor basins. Parameters used: N=1000, dt=0.2, total run time T=2000.

We study a continuous-state (graded-response) associative memory network of Amari–Hopfield type [6, 8] augmented with a multiplicative gating mechanism. This mechanism is meant to mimic the effect of neuropeptides or neuromodulators on individual neurons [12, 13]. While the detailed action and chemical and biophysical effects of these molecules are highly varied [19, 43, 44], we adopt a minimally simplified model. We focus on the modulation of neuronal conductivity, integration timescales and the neuron input-output function, and ignore neuromodulatory effects on synaptic parameters that have been explored elsewhere [45]. As the release of neuropeptides requires neuronal activity [46, 47], we couple the strength of neuromodulatory effects to the activity in the neuronal layer. Also, following the extensive neuropeptide characterization in model organisms like *C. elegans* [15, 16], we assume a varied collection of neuropeptides that can be emitted at a source neuron and influence the conductivity properties of a target neuron. These minimal assumptions are reflected in our model which consists of two recurrently connected layers (see Fig.1(b) (Left) for a schematic): the first layer of N neurons with activity (in spiking models: firing rate) x(t) and the second layer of N auxiliary units z(t) that mimic neuropeptides or neuromodulators.

Both state variables are real-valued functions of time t≥0, that is, x, $z:{\mathbb{R}}_{\geq 0}\to {\mathbb{R}}^{N}$. The neuronal layer is modeled as a fully connected (all-to-all) recurrent network with symmetric Hebbian couplings [7, 48]

(1) $$J_{ij}=\sum_{\mu =1}^{P}\xi_{i}^{\mu }\xi_{j}^{\mu },$$

where each pattern $\xi^{\mu }\in {\mathbb{R}}^{N}$ represents a memorized configuration in the neuronal layer, and P is the number of stored patterns. The memory load α=P/N determines the rank of the coupling matrix ${\left(J_{ij}\right)}_{1\leq i,j\leq N}$; in particular, for α<1 the matrix is of low-rank (at most P) [49], positioning this system within the broader class of low-rank RNNs [50]. Throughout this work, we focus on order-one loads, α=O(1). Roman letters (i,j,…∈{1,…,N}=:[N]) index neurons and neuromodulators, and Greek letters (μ,ν,…∈[P]) index stored patterns.

The dynamics of the neurons follow the equation:

(2) $$\partial_{t}x_{i}=\sigma \left(z_{i}\right)\left[-x_{i}+\frac{g}{\sqrt{PN}}\sum_{j=1}^{N}J_{ij}\phi \left(x_{j}\right)\right],$$

where ϕ:ℝ→(−1,1) is a bounded nonlinear activation function and g is a scalar gain. The prefactor $1/\sqrt{PN}$ in the interaction term ensures that it remains of order one in the thermodynamic limit [42]. A bounded, monotone nonlinear mapping σ:ℝ→[0,1] represents a switch-like multiplicative neuromodulatory gate. Its value is adaptively controlled by the neuromodulator $z_{i}(t)$, whose time-evolution is recursively driven by the activated neuronal output ${\left\{\phi \left(x_{j}\right)\right\}}_{j=1}^{N}$ as

(3) $$\tau_{z}\partial_{t}z_{i}=-z_{i}+\frac{1}{\sqrt{N}}\sum_{j=1}^{N}W_{ij}\phi \left(x_{j}\right),$$

where $\tau_{z}$ is the intrinsic neuromodulator release time-constant, and the neuromodulator-neuron couplings ${\left(W_{ij}\right)}_{1\leq i,j\leq N}$ are modeled as quenched random variables independent of the stored patterns.

#### 1. Functional forms and parameterization

Throughout the paper, we use the following specific choices for the functional forms: The neuronal activation and the neuromodulatory gating functions are assumed, respectively, to be ϕ(x)=tanh(x) and $\sigma (z)={\left(1+{\text{e}}^{-\gamma z}\right)}^{-1}$, where γ≥0 sets the steepness of the logistic sigmoid. We expect that the main results of the paper are robust to quantitative changes for many other common choices of monotonic, integrable functional forms. Using a nonmonotonic activation function is known to drastically alter associative memory dynamics [51, 52], and is not considered here.

With respect to model parameters, we fix the intrinsic time-constant of the neuromodulating layer and the scalar gain factor: $\tau_{z}=1$ and g=1.5. The stored patterns are taken to be independent and identically distributed (iid) random variables with zero mean and unit variance. Unless otherwise stated, we assume binary patterns $\xi_{i}^{\mu }\sim \mathrm{Unif}(\{-1,1\})$; we have checked numerically that replacing them with Gaussian patterns $\xi_{i}^{\mu }\sim N(0,1)$ leaves the results qualitatively unchanged, consistent with the universality of the spectral properties of large random Wishart matrices [53]. The neuromodulatory units are initialized with iid standard normal vector, $z(0)\sim N\left(0,I_{N}\right)$. The inter-layer interactions $\left(W_{ij}\right)$ are iid random variables $W_{ij}\sim N(0,1)$. Therefore, W is an asymmetric matrix, a standard choice in modeling random neural networks [30, 31, 54, 55].

Finally, the memory load α, gating steepness γ, and initial neural state x(0) serve as control parameters for the model defined by Eqs.(1–3). We note that for these choices, we observe *no* chaotic behavior in the dynamics.

#### 2. Theoretical and biophysical motifs

The value of the gate $\sigma \left(z_{i}\right)$ dynamically and adaptively controls the effective time-constant of the *i*^th^ neuron [30, 31]. As $\sigma \left(z_{i}\right)$ decreases, the effective integration time of the neuron becomes slower; when $\sigma \left(z_{i}\right)=0$ the state $x_{i}$ ceases to update and becomes closed. This specific gating mechanism is analogous to (one of) the canonical gating units originally introduced empirically as a core component of machine learning RNN architectures, such as Long Short-Term Memory (LSTM) [26, 27] and Gated Recurrent Unit (GRU) [28, 29], primarily to avoid vanishing/exploding gradient issues. However, Tallec and Ollivier axiomatically proved that the gating-like mechanism necessarily follows from postulating (quasi)invariance to nonlinear time transformations in the processed data [56] (see also [57, Ch.5]). Furthermore, in biological neural networks, an equivalent control over a neuron’s effective integration time-constant can arise from various biological mechanisms, such as synaptic shunting inhibition [58] and negative derivative feedback [31, 59] in addition to the neuromodulatory regulation we consider here [19, 24, 60]. In this light, the gate $\sigma \left(z_{i}\right)$ is not merely a heuristic but is theoretically mandated and biophysically grounded, positioning our framework as *a minimal model* of self-adaptively gated associative memory networks.

### B. Review of the ungated network

In the limiting case of γ=0, the gating function becomes constant, σ≡1/2, at all times regardless of z(t), reducing the model to the standard associative memory network without gating [6, 8, 42, 61]. Before analyzing the gated case, we briefly review the properties of this ungated limit to establish a baseline for comparison.

To quantify the alignment of the neuronal activations at time t with the stored patterns, we introduce the *overlaps* (also called Mattis magnetizations, by analogy with spin glasses [62–64]):

(4) $$m^{\mu }(t)=\frac{1}{N}\sum_{i=1}^{N}\xi_{i}^{\mu }\phi \left(x_{i}(t)\right)\;(\mu \in [P]),$$

which serve as macroscopic order parameters for the retrieval dynamics. We often focus on the steady-state overlaps $m_{\text{ss}}^{\mu }:=\lim_{t\to \infty }m^{\mu }(t)$ resulting from initial states close to the stored patterns. A steady-state overlap of order one, $m_{\text{ss}}^{\mu }=O(1)$, indicates successful memory retrieval, whereas $m_{\text{ss}}^{\mu }=O(1/\sqrt{N})$ indicates failure [62–64]. In the remainder of the paper—except in Sec. V, where we address the two-pattern case—we focus, without loss of generality, on the retrieval of a single target condensed pattern $\xi^{1}$. This corresponds to initial conditions where $m^{1}(0)=O(1)$ and $m^{\mu }(0)=O(1/\sqrt{N})$ for all μ≥2. Accordingly, we simply write $m(t)\equiv m^{1}(t)$, dropping the index, as the overlaps for the remaining patterns satisfy $m^{\mu }(t)=O(1/\sqrt{N})$ throughout the dynamics.

In Fig.1(a), we show steady-state overlaps computed from direct many-body simulations of Eq.(2) with γ=0 and N=1000. The equation was integrated using the Euler method with a time step dt=0.2. The network was initialized as $x(0)=c_{1}\xi^{1}+\eta$, where $\eta ∼N\left(0,I_{N}\right)$ and the coefficient $c_{1}$ is determined numerically to satisfy the target initial overlap m(0). Each pixel represents m(t=2000), a time sufficient for the system to relax to a steady state, for a given memory load α and initial overlap m(0), averaged over 100 realizations of ${\left\{\xi^{\mu }\right\}}_{\mu =1}^{P}$ and x(0). The vertical dashed line marks the *critical capacity*: for g=1.5, this occurs at $\alpha_{\text{c}}≃0.13$ [42]. In the heatmap, the domain characterized by $m_{\text{ss}}≃1$ (darkblue) defines the memory retrieval phase, where the stored pattern corresponds to a stable fixed point of the collective network dynamics. However, retrieval fails if either the memory load is too high $\left(\alpha >\alpha_{\text{c}}\right)$ or the system is initialized too far from the target pattern $\left(m(0)<m_{\text{c}}\right)$, where $m_{\text{c}}(\alpha )$ is defined by the lower boundary separating the retrieval and failure domains. Consequently, this plot effectively visualizes the empirical basin of attraction, a concept rigorously analyzed in the context of statistical neurodynamics [65, 66].

**FIG. 2.**
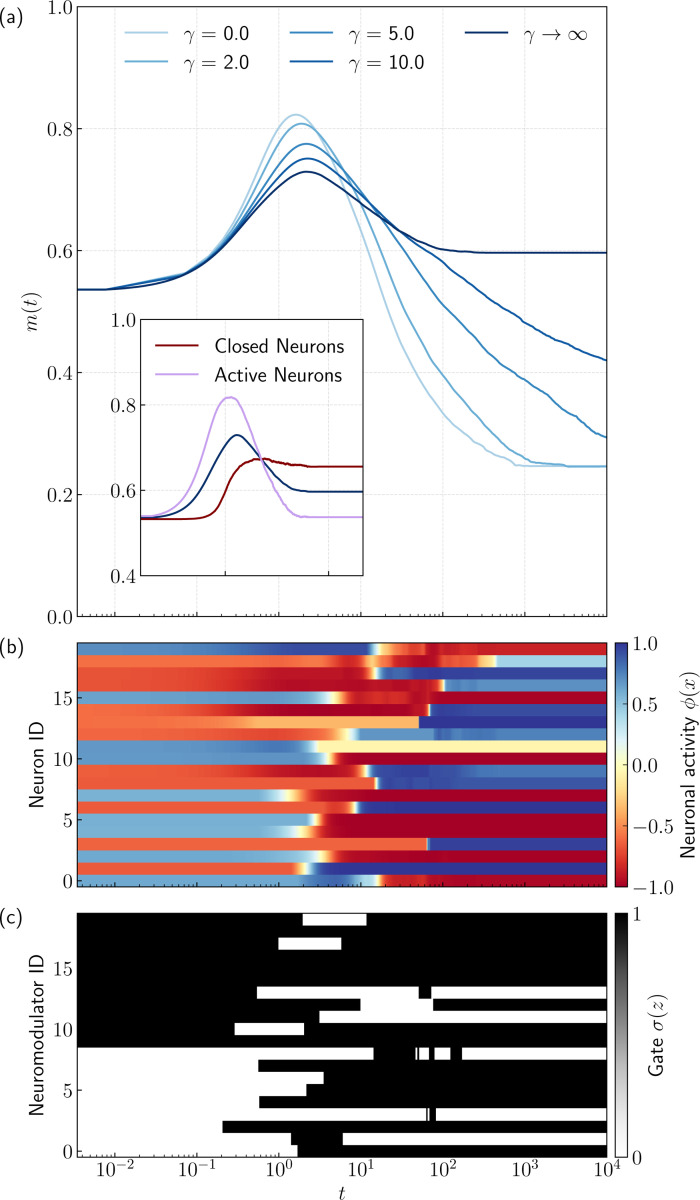
Gating stabilizes retrieval of patterns in regimes beyond critical capacity (a) The temporal evolution of the overlap m(t) in the memory overload regime $\left(\alpha =0.4>\alpha_{\text{c}}\right)$ for various gating steepness parameters γ. The case γ=0 corresponds to the standard ungated network, which exhibits a transient high-overlap state that eventually decays. As γ increases, the lifetime of this transient extends, eventually leading to a stable retrieval state. Each curve is averaged over 30 independent simulation runs with different realizations of the patterns ${\left\{\xi^{\mu }\right\}}_{\mu =1}^{P}$, couplings $W_{ij}$, and initial conditions z(0) and x(0) (fixed at m(0)≃0.55). (Inset) Decomposition of the overlap for the binary gated limit (γ→∞) into the closed $\left(m_{F_{t}}(t)\right)$ and active $\left(m_{A_{t}}(t)\right)$ sub-population overlaps. (Bottom) Representative dynamical traces of neuronal activity $\phi \left(x_{i}\right)$ (b) and gating values $\sigma \left(z_{i}\right)$ (c) for the binary gated case, illustrating the dynamical exchange between frozen and active populations before reaching the steady state. Parameters used: N=1000 and dt=0.2.

According to statistical physics analyses of the static energy landscape based on the replica method [62–64, 67], the region above the critical capacity is a spin-glass phase populated by metastable “spurious” minima, whose configurations coincide with none of the stored patterns; in this phase the memorized patterns are *no longer* stable attractors. However, dynamical studies indicate that even above $\alpha_{\text{c}}$, traces of the stored patterns persist in the energy landscape [42, 51, 65], remaining locally stable almost everywhere except in narrow unstable ravines (see, *e.g.*, Fig.4 in [51]). Due to this complex residual structure, which is reminiscent of “ghost” attractors [68–70] (*i.e.*, regions of slow dynamics emerging amidst the ruins of a destabilized fixed point), even when retrieval ultimately fails, the system trajectory typically approaches the target pattern and transiently lingers in its vicinity with an order-one overlap (as illustrated by the γ=0 curve in Fig.2(a)). This *transient retrieval* feature [42, 51, 65], often overlooked in static spin-glass analyses, suggests that associative memory dynamics possesses a typical optimal readout time [42], or can even be fundamentally improved by driving the network dynamics out of equilibrium, as exemplified by networks with nonmonotonic activation functions [51, 52, 71].

In the subsequent sections, we show that a gated network governed by Eqs.(1–3) can bypass this sharp order–to–spin-glass transition, extending the $m_{\text{ss}}=O(1)$ retrieval phase beyond the standard critical capacity threshold $\alpha_{\text{c}}$.

## III. MAIN RESULTS

In this section, we present the memory retrieval properties of gated networks obtained from direct numerical simulations of the many-body dynamics governed by Eqs.(1–3). We begin in Sec.IIIA by establishing the steady-state retrieval limits, demonstrating how the system circumvents catastrophic failure typical of overloaded networks. Next, in Sec.IIIB, we uncover the microscopic origin of this enhancement by analyzing the temporal trajectories, showing how the gate stabilizes otherwise transient dynamics. These numerical findings are subsequently corroborated in Sec.IV using Dynamical Mean-Field Theory (DMFT) using the bipartite cavity approach [42], which establishes that the observations made in finite-size simulations persist in the thermodynamic limit and in the long-time limit. Finally, in Sec.V, we investigate the emergent continuous multistability that naturally arises from the self-adaptive gating mechanism, visualizing the attractor landscape in a simplified setting.

### A. Extended memory retrieval phase

In the previous section, we reviewed that in the absence of gating (γ=0), the associative memory network exhibits a sharp phase transition between the memory-retrieval phase and the spin-glass phase. Using the same parameters, we examine steady-state overlaps of the gated networks in the binary gating limit (γ→∞), as shown in Fig.1(b). In stark contrast to the ungated case, the sharp, discontinuous transition where memory retrieval suddenly fails is notably absent. Instead, the gated network maintains an order-one retrieval overlap well beyond the critical capacity of the ungated network, $\alpha_{c}≃0.13$.

However, this expansion of the retrieval domain comes with a trade-off regarding retrieval fidelity. To quantify this, we plot the difference in steady-state overlaps between gated and ungated networks,

(5) $$\text{Δ}m_{\text{ss}}=m_{\text{ss}}^{\gamma \to \infty }-m_{\text{ss}}^{\gamma =0},$$

in Fig.1(c). Below critical storage capacity $\left(\alpha <\alpha_{\text{c}}\right)$, the difference $\text{Δ}m_{\text{ss}}$ is dominated by negative (red) to near-zero (white) values. This indicates that while the gated network still retrieves patterns, the gating mechanism slightly degrades the fidelity (magnitude) of the final overlap compared to the ungated baseline. Conversely, in the memory overload regime $\left(\alpha >\alpha_{\text{c}}\right)$, strongly positive (blue) $\text{Δ}m_{\text{ss}}$ dominate. Here, gating enables successful memory retrieval in regions where the ungated network fails completely (*i.e.*, where $m_{\text{ss}}^{\gamma =0}\approx 0$).

Fig.1 also shows that the critical retrieval boundary $m_{\text{c}}(\alpha )$ remains largely unchanged, indicating that this extended retrieval does not come at the expense of shrinking the empirical attractor basins. This property stands in contrast to dense associative memory models [32–37], which achieve huge capacity gains scaling algebraically in N but typically suffer from the narrowing of empirical basins as the interaction order n increases, manifested by an upward shift of the critical retrieval boundary $m_{\text{c}}(\alpha )$ [41, 42].

### B. Dynamics of pattern retrieval in the gated networks

In this section, we analyze the dynamics to gain insight into the mechanism underlying the extended memory retrieval observed in the phase diagrams. We focus on the temporal evolution of the overlap m(t) in the overload regime $\left(\alpha =0.4>\alpha_{\text{c}}\right)$, where the standard ungated network fails to retrieve. Fig.2(a) displays the trajectories of the overlap starting from an initial condition relatively close to the stored pattern (m(0)≃0.55).

For the ungated benchmark (γ=0), the dynamics exhibit the characteristic transient retrieval behavior discussed in Sec. IIB. The system is initially attracted toward the stored pattern, maintaining a high overlap for a finite duration by lingering in a region of slow dynamics [42]. However, because the pattern is not a stable fixed point at this high load, the system eventually escapes this vicinity and relaxes to a spin-glass state (a spurious attractor with small overlap).

Introducing the self-adaptive gating (γ>0) fundamentally alters this behavior. As the gating steepness γ increases, the duration of the high-overlap plateau extends significantly. The gating mechanism, particularly in the limit of sharp gating, seemingly stabilizes what was a transient state in the ungated dynamics into a true fixed point. Note that while the results presented in this section are numerical, in a subsequent section, using an asymptotic variant of dynamical mean-field theory, we show that the system does indeed find a true fixed point.

To further understand the dynamics, we consider the binary gate limit and partition the neuronal population into two distinct states where the gate is closed $\left(\sigma \left(z_{i}(t)\right)=0\right)$, and the population where the gate is open $\left(\sigma \left(z_{i}(t)\right)=1\right)$ [31]. Denoting the sets of indices for these populations as $F_{t}$ and $A_{t}$ respectively, we define the closed and active sub-population overlaps as

(6) $$m_{F_{t}}(t)=\frac{1}{\left|F_{t}\right|}\sum_{i\in F_{t}}\xi_{i}^{1}\phi \left(x_{i}\right),\;m_{A_{t}}(t)=\frac{1}{\left|A_{t}\right|}\sum_{i\in A_{t}}\xi_{i}^{1}\phi \left(x_{i}\right),$$

where $\left|F_{t}\right|$ and $\left|A_{t}\right|$ denote the number of neurons in each set at time t (with $\left|F_{t}\right|+\left|A_{t}\right|=N$). The total overlap m(t) is thus given by the weighted sum $m(t)=\rho_{F_{t}}(t)m_{F_{t}}(t)+\rho_{A_{t}}(t)m_{A_{t}}(t)$, where $\rho_{F_{t}}(t)=\left|F_{t}\right|/N$ and $\rho_{A_{t}}(t)=\left|A_{t}\right|/N$ are the respective population fractions.

The inset in Fig. 2(a) displays the temporal evolution of these sub-overlaps. Crucially, while the total overlap m(t) stabilizes, the membership of these populations is not static. As shown in Fig. 2(b,c), the two populations dynamically and adaptively exchange members over time. This indicates that stabilization of the macroscopic order parameter is mediated by switching between the closed and active neuron subpopulations during relaxation; once the dynamics has converged, both the overlap and the subpopulations become stationary.

## IV. THERMODYNAMIC LIMIT AND TIME-ASYMPTOTIC BEHAVIOR

The observations from the finite-size many-body simulations in the previous section raise the following question: Does the extended memory retrieval induced by gating persist in the thermodynamic limit and the long-time asymptotic regime? To address this question, we analyze the system using a Dynamical Mean-Field Theory (DMFT) which enables us to obtain analytical expressions based on the statistical properties of the gated networks. By taking the thermodynamic limit (N, P→∞ while keeping the ratio α=P/N as order-one O(1)), DMFT effectively maps the high-dimensional many-body dynamics onto a set of low-dimensional, self-consistent stochastic equations describing a representative single-site process for the variables x(t), z(t), and the order parameter m(t).

### A. Self-consistent DMFT equations

Extending the bipartite dynamical cavity method developed for ungated networks in Ref.[42] (see SM [72] for details), we obtain the effective equations of motion:

(7) $$\partial_{t}x=\sigma (z)\left[-x+\frac{g}{\sqrt{\alpha }}\left(\xi m+\eta_{x}+\alpha h_{\text{ret}}(t)\right)\right],$$

where the retarded self-interaction term is given by $h_{\text{ret}}(t)=\int duG(t,u)\phi (u)$ with shorthand ϕ(t)=ϕ(x(t)), and

(8) $$\partial_{t}z=-z+\eta_{z}.$$

Here, $m(t)=\langle \xi \phi (t)\rangle$ is the overlap of the condensed pattern, $\eta_{x}(t)$ and $\eta_{z}(t)$ are temporally colored Gaussian noises with zero mean and variances given by

(9) $$\langle \eta_{x}(t)\eta_{x}\left(t^{\prime }\right)\rangle =\alpha \int du\int du^{\prime }G(t,u)C_{\phi }\left(u,u^{\prime }\right)G\left(t^{\prime },u^{\prime }\right)$$

(10) $$\langle \eta_{z}(t)\eta_{z}\left(t^{\prime }\right)\rangle =C_{\phi }\left(t,t^{\prime }\right).$$

Finally, the self-consistency loops are closed by the two-time correlation function

(11) $$C_{\phi }\left(t,t^{\prime }\right)=\langle \phi (t)\phi \left(t^{\prime }\right)\rangle$$

and the (retarded) response functions

(12) $$R\left(t,t^{\prime }\right)=\frac{\delta \langle \phi (t)\rangle }{\delta h_{x}\left(t^{\prime }\right)}\;\text{and}\;G\left(t,t^{\prime }\right),$$

which obey causality (*i.e.*, $R\left(t,t^{\prime }\right)=G\left(t,t^{\prime }\right)=0$ for $t<t^{\prime }$), where the latter is defined via the equation

(13) $$\partial_{t}G\left(t,t^{\prime }\right)=\delta \left(t-t^{\prime }\right)+\int du\;R(t,u)G\left(u,t^{\prime }\right).$$

Here $h_{x}(t)$ represents the auxiliary external perturbation field, which is introduced to define the response function and is eventually taken to zero. The delta function in Eq. (13) represents a temporally local perturbation. The DMFT averages $\langle \ldots \rangle$ are taken over the distributions of the effective Gaussian noises $\left\{\eta_{x},\eta_{z}\right\}$, the local pattern variable ξ, and initial configurations {x(0),z(0)}. Note that the statistics of these Gaussian noises implicitly capture the ensemble averages over the quenched disorder, including the neuromodulatory couplings $\left(W_{ij}\right)$ and the non-condensed patterns.

This closed set of equations (7–13) defines the non-Markovian process governing the gated associative memory network in the thermodynamic limit. Because the order parameters appear within the dynamical expressions, these equations must be solved self-consistently (typically via numerical iteration [73, 74]) until the trajectories converge within a specified tolerance.

### B. Results and Numerical solution of DMFT

**FIG. 3.**
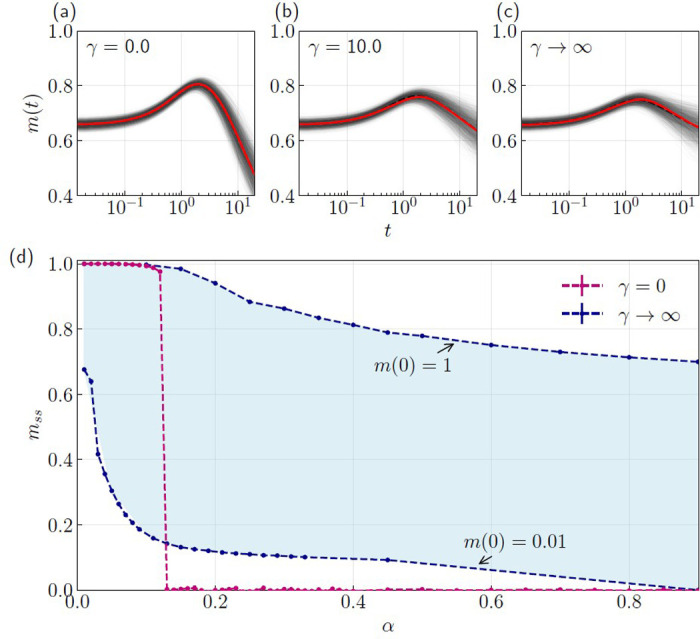
DMFT dynamics and their time-asymptotic solutions. (Top row) Overlap dynamics m(t) of ungated and gated networks for memory load above critical capacity α=0.4 and initial overlap m(0)≃0.65 using many-body simulations and DMFT: (a) ungated γ=0, (b) intermediate gating γ=10.0, and (c) binary gating γ→∞. Light gray curves correspond to individual many-body simulations while the dashed black curves are their average. Solid red curves show the DMFT predictions. (d) Time-asymptotic DMFT solutions for overlaps in ungated (red dots and dashed lines) and gated (blue dots and dashed lines) networks. The band of steady state overlap values for the gated network is bound by the the maximum and minimum steady state overlap values of the closed neurons $m_{F_{t}}^{\text{ss}}$. The maximum and minimum values were obtained from many-body simulations with m(0)=1 and m(0)=0.01, respectively, for a range of memory loads α. DMFT curves in the top row were calculated using the numerical parameters : M=5000, α=0.4, dt=0.02. In (d), dashed lines were added to guide the eye.

Our DMFT formulation for the gated network agrees well with numerical simulations across a broad range of sigmoid slopes, as shown in Fig. 3(a)–(c). This is evident as the disorder-averaged overlap m(t) from 500 many-body simulations (dashed black line) and DMFT (solid red line) fall on top of each other for all sigmoid slopes, where individual simulations are shown as light gray lines. The agreement between DMFT and numerical simulations is notable for two reasons. First, the correlations of the neuronal activity $C_{\phi }$ are non-Gaussian due to the multiplicative gating function σ(z) [30, 31], but our DMFT calculations assume they are Gaussian-distributed. The degree of non-Gaussianity of the neuron state distribution increases with steeper sigmoid functions which makes the agreement between DMFT predictions and numerical simulations for the binary gating function especially surprising. Previous work, using DMFT equations derived from the path-integral approach, showed that DMFT-predicted correlations also agreed well with numerical simulation using a sigmoid function with γ=10 [30, 31]. Secondly, our system possibly possess another noise source of non-Gaussian nature originating from uncondensed patterns for a certain memory load range. Above critical capacity for classic associative memory models, “crosstalk” or correlations between uncondensed patterns arise leading to a change in their noise distribution from Gaussian-like (below critical capacity) to non-Gaussian (above critical capacity) [75]. In fact, for discrete Hopfield networks, it was numerically confirmed that finite higher-order moments in this noise term emerge as the memory load α increases (or as m(0) decreases), potentially marking the onset of transition to the spin-glass phase [76, 77]. Nevertheless, in Fig. 3(a)–(c) we observe excellent agreement between DMFT solutions and the mean trajectories obtained from many-body simulations above critical capacity α=0.4 and for a set of different sigmoid slopes.

Having validated the DMFT equations in the dynamical regime, we now examine their asymptotic properties in the long-time limit. Due to the $O\left(T^{2}\right)$ time complexity for each iteration of the DMFT curves [42], obtaining time-asymptotic curves using the usual DMFT iteration scheme is computationally prohibitive. To this end, we derive time asymptotic DMFT equations by assuming that the dynamics reach stable fixed points $\partial_{t}x=0$, $\partial_{t}z=0$ at t→∞. This yields a set of algebraic equations that are solved self-consistently to obtain time-independent fixed-point values of the gated network’s order parameters; see SM [72] for more details.

Fig. 3(d) displays the resulting band of steady-state overlap values $m_{\text{ss}}$, calculated from the time-asymptotic DMFT expressions. Each point in the band is parameterized by an imposed overlap value specific to neurons with closed gates, specifically, their steady state overlap values $m_{F_{t}}^{\text{ss}}$. Maximum and minimum values of $m_{F_{t}}^{\text{ss}}$ will form the bounds of $m_{\text{ss}}$. We therefore constructed the bounds of Fig.3(d) by obtaining $m_{F_{t}}^{\text{ss}}$ from many-body simulations for the extreme cases of maximum initial overlap m(0)=1, and minimal overlap m(0)=0.01 for different values of memory load α. This analysis suggests that the closed-gate subpopulation neurons prevent active neurons from fully escaping the transient attractors in the landscape near the stored memory.

## V. EMERGENT CONTINUOUS MULTISTABLE ATTRACTORS AND STABILIZATION OF PATTERNS BEYOND CRITICAL CAPACITY

The observations from direct numerical simulations in Sec. III and the subsequent DMFT calculations have established that self-adaptive gating fundamentally alters memory retrieval. We can summarize the phenomenology that leads to improved recovery as follows. As in the ungated case, even above critical capacity $\alpha_{c}$, the system initially gets drawn to the memory state. In the ungated case, this transient behavior is quickly lost in the regime above critical capacity. In the gated case however, this transient increase in the overlap can be leveraged. A fraction of the neurons with improved overlap with the memory pattern can switch from active to close states in this transient regime. These neurons can provide a guiding field for the other active neurons and ensure that there are no catastrophic failures in retrieval. In the language of dynamical landscapes, this mechanism can potentially be understood as the stabilization of “ghost” attractors [68, 70]—regions of slow dynamics that persist near the stored patterns even after the true local minima have been destabilized by memory overload. This gating-induced stabilization can occur at any points in the slow regions of the original landscape; the stabilization condition can be satisfied at various points along the slow-transient trajectory. This leads to the hypothesis that a discrete fixed point of the standard ungated network can effectively be replaced by a continuously varied multiple stable fixed points. Consequently, the gated network is expected to support a continuum of final overlaps (*multistability*), where the specific fixed point reached by the dynamics depends continuously on the initial neural configurations x(0).

Evidence of this emergent multistability is indeed already apparent in the phase diagrams of Fig. 1: while the ungated network displays essentially no variation in the steady-state overlap $m_{\text{ss}}$ for a fixed load α, the gated network exhibits graded changes in $m_{\text{ss}}$, indicating a strong dependence on the initial condition m(0). Crucially, this is substantiated by the continuous band of $m_{\text{ss}}$ observed in the asymptotic DMFT analysis (Fig. 3), which implies that the gated network theoretically supports a solution space forming a continuous manifold of stable fixed points.

To obtain further intuition and to verify this hypothesis of multistability, we now visualize the attractor geometry by analyzing the phase flow in a simplified system with only two stored patterns (P=2) in the low-load limit. We set N=1000, ensuring the memory load is far below the critical capacity, and construct two orthogonal patterns: $\xi^{1}$ is sampled uniformly from ${\left\{-1,1\right\}}^{N}$, and $\xi^{2}$ is constructed by flipping the sign of exactly N/2 randomly chosen components of $\xi^{1}$. This construction guarantees that the two patterns are orthogonal to each other, satisfying $\xi^{1⊤}\xi^{2}=0$. In this setup, we track trajectories in the two-dimensional overlap phase space parameterized by $\tilde{m}=\left(m_{+},m_{-}\right)$ with $m_{\pm }=m_{1}\pm m_{2}$.

To confirm that the observed multistability is intrinsic to the gating dynamics and not an artifact of varying quenched randomness, we fix the stored patterns (defining $\left(J_{ij}\right)$), the inter-layer interactions $\left(W_{ij}\right)$, and the neuromodulatory initialization z(0). We then vary only the initial neural state x(0) (and consequently the initial overlap m(0)).

**FIG. 4.**
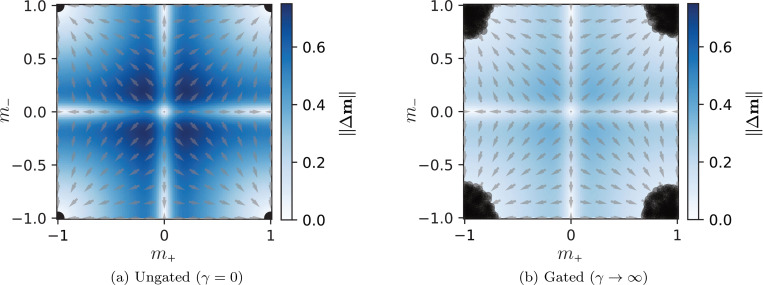
Gating creates continuous manifolds of fixed points. Flow diagrams in the overlap phase space $\tilde{m}=\left(m_{+},m_{-}\right)$, where $m_{\pm }=m_{1}\pm m_{2}$, for a network storing P=2 orthogonal patterns. Arrows represent the flow direction of the vector field, and dark circles indicate stable fixed points reached from various initial conditions. The background heatmap encodes the displacement magnitude ‖Δm‖=‖m(T)−m(0)‖ with $m=\left(m_{1},m_{2}\right)$, serving as a proxy for the attractor landscape. (a) In the ungated network, the system flows into one of four discrete fixed points (corresponding to the retrieval of $\xi^{1}$, $\xi^{2}$ or their inverses), determined solely by the initial basin of attraction. (b) In the gated network, the system reaches a continuum of distinct fixed points depending on the specific initial overlap, creating a “cloud” of stable fixed points. For both panels, the realization of patterns $\left\{\xi^{1},\xi^{2}\right\}$, couplings $\left(W_{ij}\right)$, and initialization z(0) are identical and fixed; only x(0) varies. Parameters used: N=1000, dt=0.25, and T=2000.

In the ungated network (Fig. 4(a)), the overlaps invariably converge to one of four discrete stable fixed points: $m_{\text{ss}}≃(\pm 1,0)$ or (0,±1) (equivalently ${\tilde{m}}_{\text{ss}}≃(1,\pm 1)$ or (−1,±1)), consistent with the ${\mathbb{Z}}_{2}$ equivariance of the dynamical equation (2) (reflecting the symmetry between retrieving $\xi^{\mu }$ and $-\xi^{\mu }$). Which of these fixed points the system flows into is determined solely by the attractor basin in which the initial overlap m(0) resides; for example, trajectories initiated at distinct points, such as m(0)=(0.4,0) (equivalently $\tilde{m}(0)=(0.4,0.4)$) and m(0)=(0.6,0.3) (equivalently $\tilde{m}(0)=(0.9,0.3)$), both converge to the same fixed point $m_{\text{ss}}=(1,0)$. In stark contrast, the flow in the gated network (Fig. 4(b)) reaches a continuous range of fixed points depending on m(0), forming a “cloud” of multistable attractors. Consequently, the two initial conditions m(0)=(0.4,0) and m(0)=(0.6,0.3) settle into distinct fixed points. While the specific geometry of this attractor manifold reflects the particular realization of the quenched disorder (J,W,z(0)), the existence of continuous multistability is a robust feature of the gating mechanism. In Fig. 4, the background heatmap encodes the magnitude of the displacement, ‖Δm‖=‖m(T)−m(0)‖. Thus, even without a presupposed global potential (or Lyapunov function) for the gated dynamics, this displacement metric serves as an effective proxy for the attractor landscape. This observation suggests that in the gated networks, the steady-state overlaps can be continuously controlled by choosing a different initial neural condition.

Lastly, we comment on a conceptual parallel with previous studies on *three-body interactions*, where emergent multistability was observed in coupled phase oscillators [78] and corresponding associative memory networks [79]. In those frameworks, the continuous manifold of attractors arises from specific static higher-order couplings. In contrast, our results demonstrate that similar continuous multistability can emerge dynamically from multiplicative gating applied to standard pairwise interactions. Nevertheless, we anticipate that the gating mechanism inherently induces a certain type of effective higher-order interactions. This can be seen by considering the adiabatic limit $\tau_{z}\to 0$ [80] for a network with finite γ, where the neuromodulatory layer reacts instantaneously to the neuronal layer: $z_{i}(t)≃\frac{1}{\sqrt{N}}\sum_{j}W_{ij}\phi \left(x_{j}\right)$. Substituting this into the Taylor expansion of the gating function $\sigma \left(z_{i}\right)$ around zero, the effective dynamics of $x_{i}$ becomes

$$\begin{array}{l} \partial_{t}x_{i}≃\left(\frac{1}{2}+\frac{\gamma }{4}z_{i}+O\left(z_{i}^{3}\right)\right)\left[-x_{i}+\frac{g}{\sqrt{PN}}\sum_{k}J_{ik}\phi \left(x_{k}\right)\right]\\ \;\sim \cdots +\frac{\gamma g}{4\sqrt{P}N}\sum_{j,k}W_{ij}J_{ik}\phi \left(x_{j}\right)\phi \left(x_{k}\right)+\cdots . \end{array}$$

The cross-term $W_{ij}J_{ik}\phi \left(x_{j}\right)\phi \left(x_{k}\right)$ effectively acts as a three-body interaction involving neurons i, j, and k. While this expansion formally diverges in the binary limit (γ→∞), it qualitatively illustrates how multiplicative gating generates a hierarchy of higher-order correlations. Elucidating the formal relationship between these dynamically generated interactions and explicit higher-order couplings remains an intriguing direction for future work.

## VI. CONCLUSION AND OUTLOOK

In this work, we demonstrated that a minimal model of self-adaptive gating fundamentally enhances the classical associative memory properties. Specifically, we uncovered three key insights regarding the impact of gating on memory retrieval properties: First, the gated network circumvents the catastrophic spin-glass transition, maintaining high-fidelity retrieval well beyond the standard critical capacity without shrinking the empirical basins of attraction. Second, we identified the dynamical origin of this enhancement as the gating-induced stabilization of transients, where the adaptive freezing of neuronal integration times stabilizes the slow regions of dynamics near the stored patterns converting them into stable fixed points. Third, the stabilization of transients result in the emergence of continuous multistability, transforming the attractor landscape from a discrete fixed point into a continuous manifold of stable fixed points. All these findings from direct many-body numerical simulations were corroborated by DMFT, establishing that these phenomenologies are genuine features of gating in the thermodynamic limit and in the long-time limit, rather than artifacts of finite system size or long-lived transient effects.

Having established and theoretically verified the effects of simple neuromodulation on memory retrieval, a natural question arises, *how applicable are the findings of this minimal model for biological systems?* Across all species investigated, the neuropeptide repertoire is vast, with multiple neuron classes releasing multiple neuropeptides. This sets up an impressive layered complexity, potentially adding substantial computational capacity to small nervous systems such as in nematodes. Even mammalian brains show remarkable diversity of neuropeptide expression, whose local release and uptake affect local neuronal populations. Experimentally, it is hard to assess the impact of these modulators in complex brains, as spatial control, or the ability to image the modulators directly are very limited. Computational work suggests that further efforts in this direction are worthwhile - these modulators may prove to be essential for capturing key aspects of brain function. Indeed, dopamine (a neuromodulator) has been found to lead to increased working memory in the hippocampus by affecting individual neurons in the region [81]. Neuropeptides have been shown to prolong and stabilize behavioral states by signaling specific neurons in *C. elegans* [82, 83]. Furthermore, neuropeptides were reported as necessary for line attractor neuronal activity dynamics in mice behavior [22].

While each of the previous examples highlight features potentially related to our findings, nonetheless, our gated RNN should be improved for modeling biological systems. Specifically, the neuromodulator-neuron couplings $W_{ij}$ are currently quenched random variables, but based on the neuropeptidurgic connectome of *C. elegans* [15], they should contain some structure and connect specific neuron populations per neuropeptide. One path towards this would be to learn $W_{ij}$ couplings from measurements of neuronal activity. In particular, *C. elegans* would be good candidate for this due to its small set of neurons and its extensive publicly available data covering the activity of the majority of its neurons [2, 3, 84, 85]. Additionally, the model could be adapted for spiking networks as it currently uses a continuous state akin to analog neurons or as a proxy for firing rates.

Furthermore, the emergence of continuous multistable attractors from gating indicates the possibility of generalization using gated RNNs. In fact, Costacurta et al. have shown the appearance of generalization in low-rank gated RNNs which were trained for temporal tasks [86]. The gating layer of their trained network was essential for the correct classification of inputs that were outside of their training parameter range. Gating, in general, seems to be not only beneficial in biological systems but also ubiquitously useful in machine learning, as gated large language models have been shown to improve their expressivity and trainability [87], and an interesting direction would be the effects of gating on generalization in gated diffusion models and other forms of neuromorphic computing.

### Note added during manuscript preparation:

During the final preparation of this manuscript, a relevant preprint appeared [88] that also concerns memory retrieval above the traditional critical capacity. While both studies employ two-layer network architectures, the mathematical formulations and physical interpretations differ fundamentally. In our model, the self-adaptive gating mechanism acts *multiplicatively* on the dynamics (modulating the effective time-constants), whereas the model in Ref. [88] employs an *additive* modulation of the synaptic interactions. Consequently, these approaches map to distinct biophysical motifs: our framework models neuromodulation (specifically, neuropeptidergic regulation), whereas their framework models short-term synaptic plasticity. Furthermore, the underlying mechanisms are of a distinct nature; while Ref. [88] proposes a “trampoline” mechanism with underlying Lyapunov structure, our work identifies gating-induced stabilization of ghost-like transients—accompanied by emergent multistability—as the novel physical perspective driving extended retrieval without assuming a Lyapunov function.
